# Euphocactoside, a New Megastigmane Glycoside from *Euphorbia cactus* Growing in Saudi Arabia

**DOI:** 10.3390/plants11060811

**Published:** 2022-03-18

**Authors:** Hanan Y. Aati, Shagufta Perveen, Jawaher Al-Qahtani, Jiangnan Peng, Areej Al-Taweel, Ali S. Alqahtani, Ali ElGamal, Giuseppina Chianese, Fahd A. Nasr, Orazio Taglialatela-Scafati, Mohammad K. Parvez

**Affiliations:** 1 Department of Pharmacognosy, College of Pharmacy, King Saud University, Riyadh 11495, Saudi Arabia; jalqahtani@ksu.edu.sa (J.A.-Q.); amaltaweel@ksu.edu.sa (A.A.-T.); alalqahtani@ksu.edu.sa (A.S.A.); aelgamer@ksu.edu.sa (A.E.); fnasr@ksu.edu.sa (F.A.N.); mohkhalid@ksu.edu.sa (M.K.P.); 2 Department of Chemistry, School of Computer, Mathematical and Natural Sciences, Morgan State University, Baltimore, MD 21251, USA; jiangnan.peng@morgan.edu; 3 Department of Pharmacy, School of Medicine and Surgery, University of Naples Federico II, Via Montesano 49, 80131 Naples, Italy; g.chianese@unina.it (G.C.); scatagli@unina.it (O.T.-S.)

**Keywords:** *Euphorbia cactus*, euphocactoside, megastigmane glycoside, ellagic acid glycoside, flavonoids, cytotoxic activity

## Abstract

A phytochemical investigation of the aerial parts of *Euphorbia cactus* Ehrenb. ex Boiss. revealed a new megastigmane, euphocactoside (**5**), along with eleven known metabolites. Euphocactoside (**5**) is the 3-*O*-glucoside derivative of a polyhydroxylated megastigmane showing unprecedented structural features. The structure of euphocactoside, including stereochemical details, was elucidated by extensive spectroscopic analysis based on 1D and 2D nuclear magnetic resonance (NMR) and high-resolution mass spectrometry (HR-ESIMS). The isolated compounds were evaluated for their cytotoxic activity against three different human cancer cell lines, namely, A549 (lung), LoVo (colon), and MCF-7 (breast), using MTT assay, and moderate to marginal activities were observed for compounds **1**–**3**, **8** and **9** against all three cell lines.

## 1. Introduction

The genus *Euphorbia* (family Euphorbiaceae) is classified as the third largest genus of angiosperms and includes more than 2000 species of flowering plants with different shapes, varying from creeping herbs to shrubs and trees [1]. Several members of this genus, especially those living in arid lands of Africa, India, and the Arabian Peninsula, are spined succulents that resemble cactus plants but, differently from cacti, euphorbias possess a milky exudate, often poisonous sap [1].

The aerial parts, especially the latex, of *Euphorbia* plants are known to contain several classes of interesting secondary metabolites, such as phenolics (including ellagic acid lactones), triterpenes, flavonoids, and coumarins [2]. The triterpenoids belonging to the families of euphol and euphorbol, typical of this genus, have been found to possess several interesting bioactivities [3]. However, the most representative and diverse class of *Euphorbia* secondary metabolites are undoubtedly diterpenes. A wide array of typical structural frameworks, from macrocyclic to polycyclic architectures, have been identified, including tiglianes, ingenanes, daphnanes, lathyranes, jatrophanes, and myrsinanes [4]. These compounds have been identified as the components mainly responsible for the potent skin-irritating and tumor-promoting effects associated with *Euphorbia* latex [5]. However, the biological activities ascribed to these metabolites also have the potential to be translated into interesting pharmacological activities effective in curing illnesses that affect human health [6]. The best example is probably ingenol metabutate (Picato^®^, LEO Pharma, Copenhagen, Denmark), a diterpene isolated from *E. peplus*, which has been approved for the treatment of actinic keratosis by the FDA [7]. Other potential applications include the analgesic activity of the TRPV1-stimulating resiniferatoxin [8,9] and the multi-drug resistance inhibition on cancer and fungal cells of jatrophanes [10,11,12].

*Euphorbia cactus* Ehrenb. ex Boiss. is a perennial herbaceous plant with a milky latex in the aerial parts and roots. It is native to Eritrea, Ethiopia, and the Arabian Peninsula, with an especially high distribution in the southern region of Saudi Arabia, particularly in the Fayfa mountains. Although this species is known to the local populations for its curative properties for many diseases, and, in particular, the aerial parts and flowers are used as wound healing agents [13], to our knowledge, its detailed phytochemical profile is still lacking in the scientific literature. The only report on Saudi *E. cactus* described promising anticancer, antioxidant, and antimicrobial effects of the methanolic extract [14]. Anticancer activity has also been reported for extracts of *E. cactus* of different origin [13]. In 2018, Al-Hajj et al. reported on the antileishmanial activity of latex extracts of a Yemeni *E. cactus* and suggested the usefulness of these extracts as an alternative remedy in curing cutaneous leishmaniasis [15].

**Figure 1:**
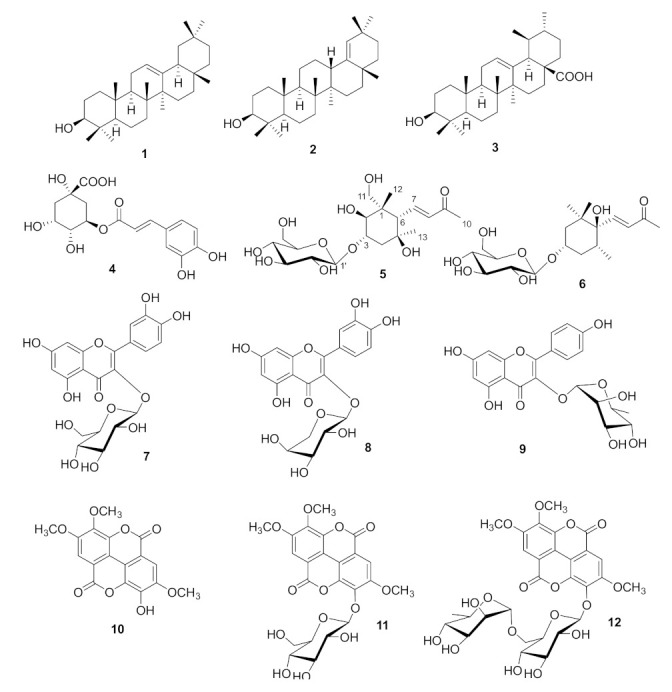
Chemical structures of metabolites isolated from *E. cactus*.

The present investigation was aimed at filling the gap of knowledge on the phytochemical composition and/or isolation of the active principles from Saudi *E. cactus*. From the aerial part of this plant, we isolated twelve secondary metabolites (**1**–**12**, Figure 1), including an unprecedented megastigmane glucoside, named euphocactoside (**5**). In this manuscript, we describe the isolation and structural identification of these compounds, as well as the evaluation of their cytotoxic effect on three human cancer cell lines.

## 2. Results and Discussion

Aerial parts of *E. cactus* were extracted with ethanol, and the obtained gummy material was suspended in water and partitioned with dichloromethane and then *n-*butanol. The organic fractions were chromatographed on silica gel and then further separated using repeated column and HPLC fractionations to obtain twelve pure compounds (**1**–**12**, Figure 1). These included the known triterpenoids β-amyrin (**1**), 3-hydroxy-olean-18-ene (**2**), ursolic acid (**3**) [16,17], and chlorogenic acid (**4**) [18]; two megastigmane glucosides, namely, the new euphocactoside (**5**) and the known camellistigoside A (**6**), previously reported only from the non-euphorbiaceous plant *Camellia bugiamapensis* [19]; three flavonoid glucosides, namely, quercetin-3-*O*-β-D-glucoside (**7**) [20], quercetin-3-*O*-β-arabinopyranoside (**8**) [21], and kaempferol 3-*O*-α-rhamnoside (**9**) [22]; and three ellagic acid derivatives, namely, 3,4,3’-tri-*O*-methyl ellagic acid (**10**), 3,4,3’-tri-*O*-methyl ellagic acid 4′-glucopyranoside (**11**), and 3,4,3’-tri-*O*-methyl ellagic acid 4’-rutinoside (**12**) [23]. All these compounds were identified by comparing their spectroscopic data with those reported in the literature. It is worth noting that *E. cactus aerial* part extracts did not show detectable amounts of diterpenoids, and this finding can be explained by considering the secondary metabolites diversity of Euphorbia species depend on collection time and geographic regions, as recently evidenced by Ernst et al. [24].

The molecular formula of euphocactoside (**5**) was determined to be C_19_H_32_O_10_, indicating four unsaturation degrees, based on positive ions HR-ESIMS data (*m*/*z* 443.1899 [M + Na]^+^, calcd for C_19_H_32_O_10_Na, 443.1893). The ^1^H NMR spectrum of **5** (in DMSO-*d*_6_, Table 1, Figure S1) showed signals for three methyl singlets (δ_H_ 0.95, 1.11, and 2.27); two low-field multiplets attributable to olefinic methines (δ_H_ 6.25, and 6.73); two oxymethines; and signals of a hexopyranose, including a β-anomeric proton resonating at δ_H_ 4.25, d, *J* = 8.0 Hz). The ^13^C NMR spectrum of **5** (Table 1, Figure S2) was analyzed with the aid of a DEPT135 experiment, which sorted the nineteen carbon resonances into three methyls, three methylenes (two oxygenated), ten methines (two s*p*^2^ carbons and eight *sp*^3^, seven of which were oxygenated), and three unprotonated carbons (δ_C_ 82.4 and 198.0, the latter attributable to a ketone carbonyl), Figure S3.

**Figure 2:**
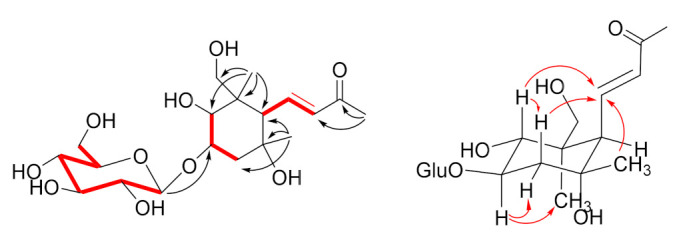
(**Left**): COSY (red bold) and HMBC (black arrows) correlations; (**right**): ROESY (red arrows) correlations of compound **5**.

The two-dimensional NMR COSY spectrum allowed for the definition of three spin systems (Figure 2 and Figure S4), namely, the hexopyranose and two three-carbons moieties, the first including the two *trans*-oriented olefinic methines (*J* = 15.6 Hz) and an allylic methine, and the second connecting two oxymethines and an *sp*^3^ methylene. Having associated all the proton signals with those of the directly attached carbon atoms through the HSQC experiment, we could use the HMBC spectrum to connect the above-defined moieties (Figure 2, Figures S5 and S6). The deshielded methyl singlet at δ_H_ 2.27 showed cross-peaks with the ketone carbonyl (C-9) and with the olefinic C-8 (δ_C_ 135.7). The methyl singlet at δ_H_ 0.95 correlated with the allylic C-6 (δ_C_ 59.5), the unprotonated C-1 (δ_C_ 49.6), the oxymethine C-2 (δ_C_ 73.0) and the oxymethylene C-11 (δ_C_ 72.9). Similarly, the methyl singlet at δ_H_ 1.11 correlated with C-6, the oxygenated quaternary carbon C-5 (δ_C_ 82.4), and the methylene C-4 (δ_C_ 40.2). In this way, the aglycone part was clearly arranged into a 2,3,5,11-tetrahydroxylated megastigmane unit. The sugar unit was easily identified as a β-glucopyranose on the basis of the large proton–proton coupling constants, pointing to an axial orientation of H-1 to H-2 protons, and of the characteristic ^13^C NMR values [19]. This sugar unit was confidently attached at C-3 of the megastigmane core based on the HMBC cross-peak of the anomeric proton H-1 with C-3 (δ_C_ 81.3). In this way, the planar structure of euphocactoside (**5**) was completely defined, but the relative stereochemical arrangement of the five adjacent stereogenic carbons belonging to the six-membered carbocyclic ring still needed to be elucidated. The *trans*-diaxial orientation of H-2 and H-3 was deduced from the large coupling constant (*J* = 8.3 Hz) of the corresponding protons, implying that H-1, H-2, and H-3 are all axially oriented, while the relative orientation of the remaining centers could be inferred from the 2D NMR ROESY correlations. To this aim, ROESY correlations of H-3 with H_3_-12, and those of H-2, H-4ax, and H_3_-13 with H-7 completely defined the relative stereochemistry of euphocactoside (**5**), Figure S7. The absolute configuration of **5** was not assigned, but it was drawn as that of the co-occurring camellistigoside A (**6**) at the parallel stereocenters.

Euphocactoside (**5**) is a new tetrahydroxylated megastigmane glucoside, and, to the best of our knowledge, it is the first member of this class to show an -OH group at one of the two geminal methyls attached at C-1.

The isolated compounds were evaluated for their cytotoxic activity against different human cancer cell lines, namely, A549 (lung), LoVo (colon), and MCF-7 (breast). The IC_50_ values for the isolated compounds generated from the dose–response curves are presented in Table 2. Only compounds **1**–**3** and **8**–**9** exhibited cytotoxic activity, with IC_50_ values in the range of 18–55 μM that, compared to the activity of the reference compound doxorubicin, must be considered modest. The bioactivity detected for triterpenoids **1**–**3** appears to agree with previously reported antiproliferative activities of these compounds and with a general higher potency of acidic derivatives. For example, the antiproliferative effects of β-amyrin (**1**) against hepatic carcinoma have previously been reported [25] and, similarly, ursolic acid (**3**) has shown significant cytotoxic effects in previous studies [26]. Flavonoids are well-known anticancer compounds [27]. Among the tested compounds, quercetin-3-*O*-β-arabinopyranoside (**8**) exerted the highest cytotoxic activity, with IC_50_ values below 20 µM against the three cancer cell lines. It can be anticipated that the cytotoxic mechanisms of this compound, namely, dual action against ROS, apoptosis induction on cancer cells, and their abilities to down-regulate pro-inflammatory signaling pathways, are common to other flavonoids [27]. However, the antiproliferative activity observed for compound **8** necessitates further pharmacological investigation to elucidate the exact molecular mechanism and rationalize the interesting differences in potency compared with the glucoside (**7**) and the rhamnoside (**9**) congeners, which point to a crucial role also played by the sugar unit.

## 3. Materials and Methods

### 3.1. General

A MX-500 Bruker spectrometer was used to measure one-dimensional (1D) and two-dimensional (2D) nuclear magnetic resonance (NMR) spectra. The chemical shifts (δ) were calculated (ppm) relative to TMS and J scalar coupling constants reported in Hz. MS analyses were carried out on an Agilent triple quadrupole 6410 QQQ LC/MS mass spectrometer with an ESI ion source (nebulizer gas pressure is 60 psi, gas temperature is 350 °C, and flow rate is 12 L/min), operating in the negative and positive scan modes of ionization through direct infusion method using methanol–water (4:6 *v*/*v*) at a flow rate of 0.5 mL/min. Separations and purifications of secondary metabolites were carried out by using column chromatography either on silica gel 70–230 mesh or RP-18 (E. Merck, Darmstadt, Germany). RP-18 (Merck) and pre-coated silica gel 60 F254 TLC plates were used to check the fractions, and the spots were detected by UV light and by spraying with ceric sulphate and sulfuric acid reagent followed by heating on a hot plate (TLC plate heater III CAMAG, Muttenz, Switzerland). Analytical and reagent grade solvents were obtained from Sigma-Aldrich (St. Louis, MO, USA). NMR deuterated methanol (CD_3_OD) and dimethylsulfoxide (DMSO-d_6_) were purchased from Cambridge Isotope Laboratories (Tewksbury, MA, USA).

### 3.2. Plant Material

**Figure 3:**
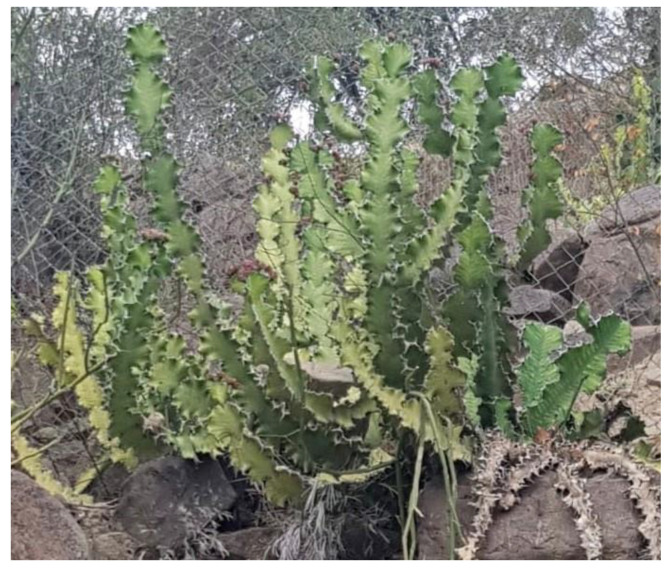
*E. cactus* aerial parts growing in Fayfa mountain, Saudi Arabia.

The aerial parts of *E. cactus* were collected in the area of the Fayfa mountains, Saudi Arabia, in December 2018, and identified by Dr. Rajakrishnan Rajagopal, taxonomist of the Science College, King Saud University. A voucher specimen (No. 24538) was deposited at the Herbarium of Science College, King Saud University, KSA (Figure 3).

### 3.3. Extraction and Isolation

The aerial parts of *E. cactus* (3.2 kg) were extracted by infusion with 95% EtOH (3 × 6.5 L) at room temperature. The collected alcoholic extract was dried using a rotary evaporator to give 98.3 g of crude gummy extract that was partitioned between water and dichloromethane (DCM, 35.1 g) and then the water residue was partitioned against *n-*butanol (61.2 g). The DCM fraction was separated using silica gel column chromatography (2.0 kg, 100 × 49 cm) eluted with a gradient mixture of DCM–MeOH (95:5→20:80, *v*/*v*) to yield 4 fractions (ECP1-ECP4). Fraction ECP1 (12.7 g) was subjected to silica gel CC (590 g, 60 × 35 cm) with a gradient elution of petroleum ether–EtOAc (90:5→15:85, *v*/*v*) to afford 5 subfractions (ECP1_a_–EC1_e_). Subfraction ECP1_d_ (4.01 g) was chromatographed repeatedly on silica gel (250 g, 25 × 12 cm, petroleum ether–CHCl_3_ (80:20→10:90, *v*/*v*)); subfraction ECP1_d_4 was further recrystallized using MeOH to give compound **1** (11.35 mg). Fraction ECP4 (172 mg) was purified using open column chromatography (850.3 g petroleum ether–EtOAC, 85:15→15:80, *v*/*v*) to provide 3 subfractions that were then purified via HPLC on normal phase eluting with hexane–DCM 9:1 to afford compounds **2** (10.7 mg, Rt 11.3 min) and **3** (15.3 mg, Rt 12.5 min). The butanolic fraction was chromatographed on silica gel and eluted with DCM–MeOH gradient mode (85:15→25:75, *v*/*v*) to give 6 subfractions (EC1-6). Fraction EC1 (6.2 g) was chromatographed on silica gel (300.2 g, 100 × 20 cm) with a gradient elution of DCM–MeOH (85:15→20:80, *v*/*v*) to give 8 subfractions. Subfraction EC1_c_ (2.5 g) was chromatographed on silica gel (150.23 g, 100 × 23 cm, 70) with a gradient elution of DCM–EtOAC (65:35→0:100, *v*/*v*) to give 3 subfractions that were then further purified using HPLC (hexane/EtOAc 1:1) to give **4** (10.3 mg, Rt 9.3 min). Fraction EC2 (8.5 g) was chromatographed using reversed-phase column chromatography with a gradient of H_2_O–MeOH (from 9:1 to 1:9) to afford 5 subfractions. EC2_b_ (1.95 g) was subjected to further purification with reversed-phase column chromatography (eluted with H_2_O–MeOH 1:1) to give euphocactoside (**5**, 10.4 mg). Subfraction EC2_e_ (3.2 g) was chromatographed on silica gel (145 g, 100 × 25 cm) with a gradient elution of DCM–MeOH (65:35→25:75, *v*/*v*) to give white crystalline compound **6** (13.3 mg). Fraction EC4 was purified using reversed-phase chromatography (10.6 g, RP-18, 150 × 20 mm) eluted with a of gradient H_2_O–MeOH (from 9:1 to 1:9) to afford compounds **7** (18.2 mg), **8** (15.4 mg), and **9** (12.3 mg). Fraction EC5 (17.3 g) was chromatographed on silica gel (1.5 kg, 100 × 25 cm) with a gradient elution of DCM–MeOH (90:10→ 10:90, *v*/*v*) to give 4 subfractions (EC5_a–d_). Subfraction EC5_b_ (3.23 g) was separated using silica gel column chromatography (120.3 mg, 90 × 2.0 cm) eluted with a MeOH–DCM gradient (from 1:9 to 9:1) to afford compounds **10** (11.8 mg) and **11** (14.3 mg). Finally, subfraction EC6 (15.0 g) was separated using column chromatography packed with normal phase silica eluted with 35% MeOH in DCM to give 3 subfractions. Subfraction EC6_b_ was recrystallized by using acetone and gave pure compound **12** (25.0 mg).

Euphocactoside (**5**). White powder; [α]_D_-11.2 (c 0.10, CH_3_OH); (+)-ESIMS *m*/*z* 443.1899 [M+Na]^+^ (calcd for C_19_H_32_O_10_Na, 443.1893); for ^1^H and ^13^C NMR data, see Table 1.

### 3.4. Cytotoxic Activity

Three different cancer cell lines, namely, A549 (lung), LoVo (colon), and MCF-7 (breast), were cultured in DMEM media (Gibco, Big Cabin, OK, USA) with 10% fetal bovine serum at 37 °C in a humidified atmosphere containing 5% CO_2_. Cell viability was measured by MTT assay as previously described [28]. In brief, the cells were placed in a 96-well plate at a density of 5 × 10^4^ cells/mL and treated with compounds at different concentrations (0, 3.125, 6.25, 12.5, and 25 µg/mL), using a DMSO solvent as a vehicle or doxorubicin as a positive control for 48 h. Then, the MTT solution (5 mg/mL)/well was added to each well and incubated for 4 h. Thereafter, formazan was solubilized in isopropanol and measured spectrophotometrically at 570 nm using a microplate reader (BioTek, Shoreline, WA, USA). The results were reported as the cell viability percentage, and IC_50_ values were calculated from the dose–response curve.

## 4. Conclusions

In conclusion, a phytochemical investigation on the Saudi plant *E. cactus* revealed a peculiar secondary metabolites profile, including triterpenoids, megastigmanes, flavonoid glycosides, ellagic acid derivatives, and a lack of diterpenoids. The new tetrahydroxylated megastigmane glucoside euphocactoside (**5**) was isolated and fully characterized. This class of compounds is not unprecedented in *Euphorbia* plants, but it is also not very common since less than a dozen examples are reported in the literature. Euphocactoside (**5**) innovates the structural diversity associated with this class of metabolites, being the first example to show a free hydroxyl group linked to one of the two geminal methyls at position 1. The antiproliferative potential previously reported for this plant [13] can be at least in part ascribed to the triterpenoid and flavonoid glucoside content, with a significantly higher potency shown by quercetin-3-*O*-β-arabinopyranoside, which is worthy of further investigation.

## Figures and Tables

**Table 1:** ^1^H- and ^13^C NMR data for euphocactoside (**5**) in DMSO-*d*_6_.

Position	δ_H_ (mult., *J* in Hz)	δ_c_
1	-	49.6
2	3.51 (d, 8.3)	73.0
3	3.68 (ddd, 10.0, 8.3, 6.5)	81.4
4a	2.01 (dd, 13.0, 6.5)	40.2
4b	1.67 (dd, 13.0, 10.0)	
5	-	82.4
6	2.36 (d, 10.2)	59.5
7	6.73 (dd, 15.6, 10.2)	141.6
8	6.25 (d, 15.6)	135.7
9	-	198.0
10	2.27 (s)	27.9
11°	3.91 (d, 8.0)	72.9
11b	3.26 (d, 8.0)	
12	0.95 (s)	18.3
13	1.11 (s)	24.6
1′	4.25 (d, 8.0)	102.8
2′	3.00 (dd, 8.0, 7.8)	70.7
3′	3.17 (t, 7.8)	77.3
4′	3.39, overlapped	77.0
5′	3.35, overlapped	73.6
6′a	3.71, m	61.6
6′b	3.41, overlapped	

**Table 2:** Cytotoxic effects of isolated compounds against three cancer cell lines ^1^.

Compound	Cell Lines and IC_50_ (µM)		
	A549	LoVo	MCF-7
**1**	49.9 ± 2.7	34.4 ± 1.2	36.3 ± 1.8
**2**	53.5 ± 2.4	37.2 ± 1.3	46.1 ± 1.2
**3**	26.5 ± 0.4	26.4 ± 0.5	31.9 ± 2.9
**4**	NA	NA	NA
**5**	NA	NA	NA
**6**	NA	NA	NA
**7**	NA	NA	NA
**8**	19.8 ± 0.3	19.0 ± 0.4	18.6 ± 0.3
**9**	52.4 ± 0.7	46.3 ± 0.4	52.7 ± 0.6
**10**	NA	NA	NA
**11**	NA	NA	NA
**Doxorubicin**	3.2 ± 0.3	4.74 ± 0.2	5.0 ± 0.7

^1^ Duration of the treatment = 48 h. Values are the mean ± SD (*n* = 3); NA = no activity at 25 µg/mL (highest concentration tested). IC_50_ values were calculated from dose–response curve using OriginPro 8.5 software.

## Data Availability

Not applicable.

## Supplementary Materials

The following supporting information can be downloaded at: https://www.mdpi.com/article/10.3390/plants11060811/s1, Figure S1: 1H NMR spectrum (DMSO-d6) of euphocactoside (**5**), Figure S2: 13C NMR spectrum (DMSO-d6) of euphocactoside (**5**), Figure S3: DEPT135 NMR spectrum (DMSO-d6) of euphocactoside (**5**), Figure S4: COSY NMR spectrum (DMSO-d6) of euphocactoside (**5**), Figure S5: HMBC NMR spectrum (DMSO-d6) of euphocactoside (**5**), Figure S6: HSQC NMR spectrum (DMSO-d6) of euphocactoside (**5**), Figure S7: ROESY NMR spectrum (CD3OD) of euphocactoside (**5**).
