# Supplementary material for: Euphocactoside, a New Megastigmane Glycoside from Euphorbia cactus Growing in Saudi Arabia
Source: Plants (Basel). 2022 Mar 18;11(6):811. doi: 10.3390/plants11060811 (PMC8955017; doi:10.3390/plants11060811)
Supplement: Supplementary file 1 [file plants-11-00811-s001.zip › plants-1604924-supplementary.pdf]

Supplementary Material

Euphocactoside, a New Megastigmane Glycoside from *Euphorbia cactus* Growing in Saudi Arabia

Hanan Y. Aati [1,*], Shagufta Perveen [2,*], Jawaher Al-Qahtani [1], Jiangnan Peng [2], Areej Al-Taweel [1], Ali S. Alqahtani [1], Ali ElGamal [1], Giuseppina Chianese [3], Fahd A. Nasr [1], Orazio Taglialatela-Scafati [3] and Mohammad K. Parvez [1]

[1] Department of Pharmacognosy, College of Pharmacy, King Saud University, Riyadh 11495, Saudi Arabia; jalqahtani@ksu.edu.sa (J.A.-Q.); amaltaweel@ksu.edu.sa (A.A.-T.); alalqahtani@ksu.edu.sa (A.S.A.); aelgamer@ksu.edu.sa (A.E.); fnasr@ksu.edu.sa (F.A.N.); mohkhalid@ksu.edu.sa (M.K.P.)

[2] Department of Chemistry, School of Computer, Mathematical and Natural Sciences, Morgan State University, Baltimore, MD 21251, USA; jiangnan.peng@morgan.edu

[3] Department of Pharmacy, School of Medicine and Surgery, University of Naples Federico II, Via Montesano 49, 80131 Naples, Italy; g.chianese@unina.it (G.C.); scatagli@unina.it (O.T.-S.)

* Correspondence: hati@ksu.edu.sa (H.Y.A.); shagufta792000@yahoo.com (S.P.)

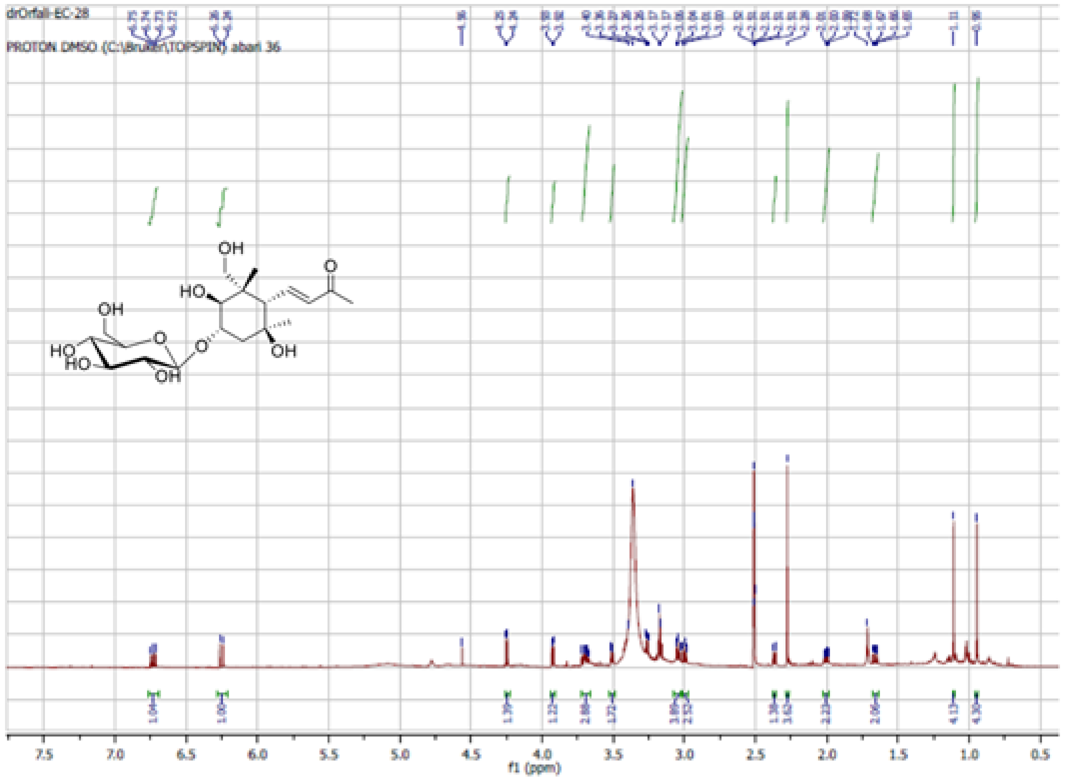

Figure S1. ^{1}H NMR spectrum (DMSO-d_6) of euphocactoside **(5).**

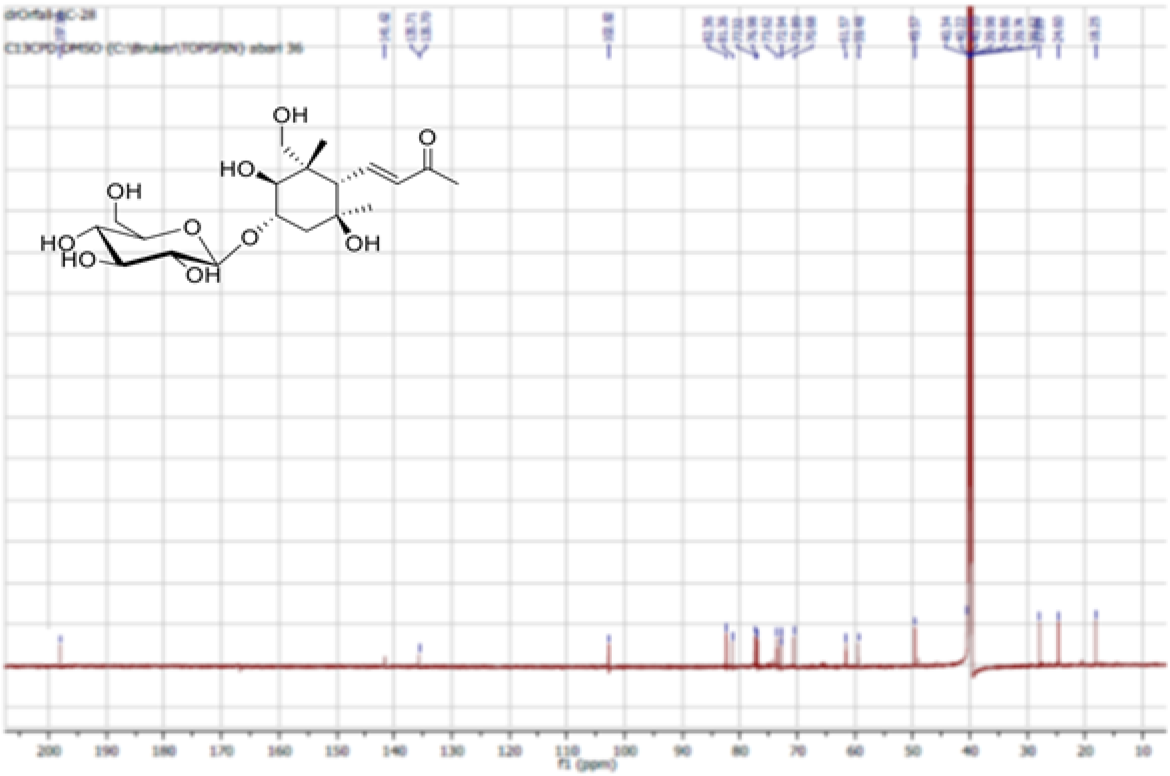

Figure S2. ^{13}C NMR spectrum (DMSO-d_6) of euphocactoside (**5**).

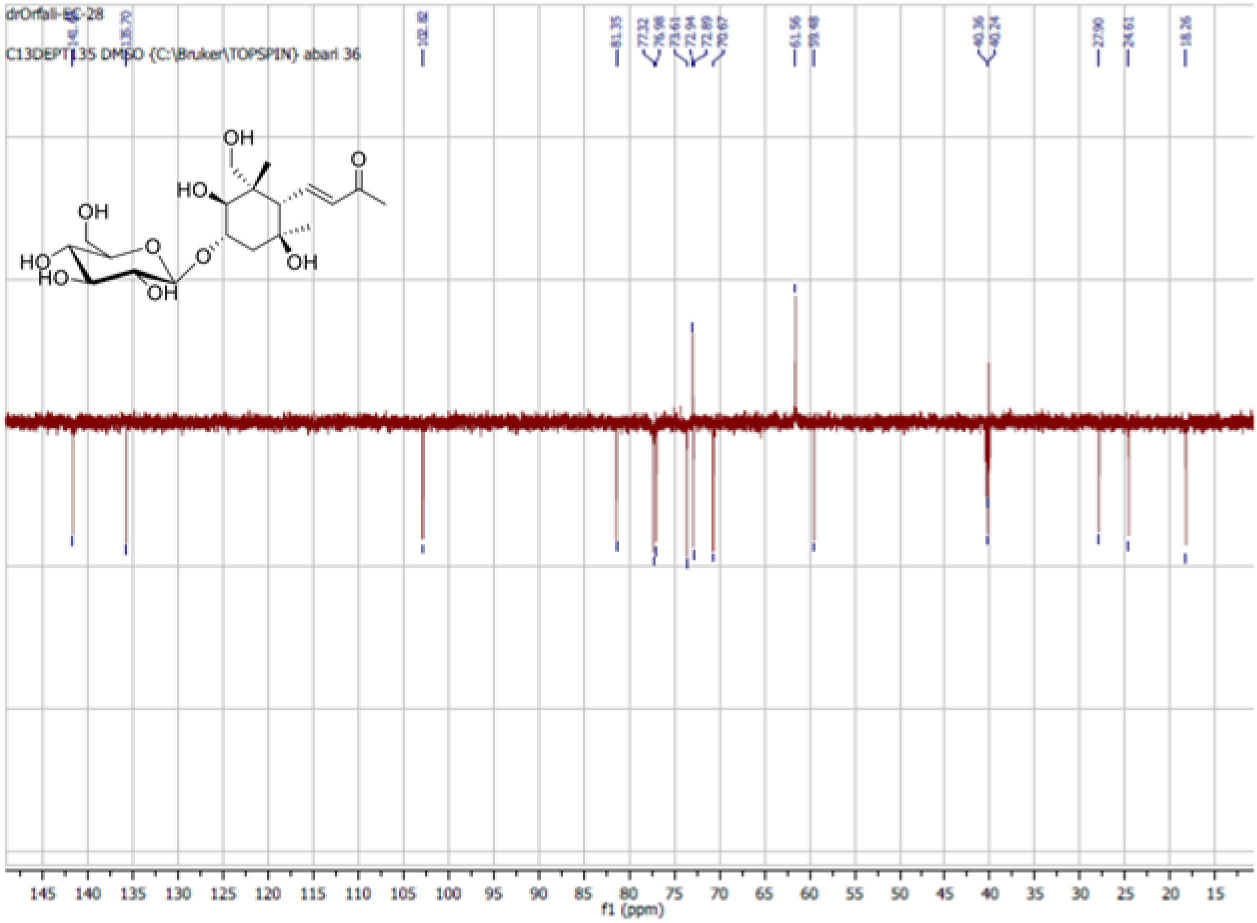

Figure S3. DEPT135 NMR spectrum (DMSO-d_6) of euphocactoside (**5**).

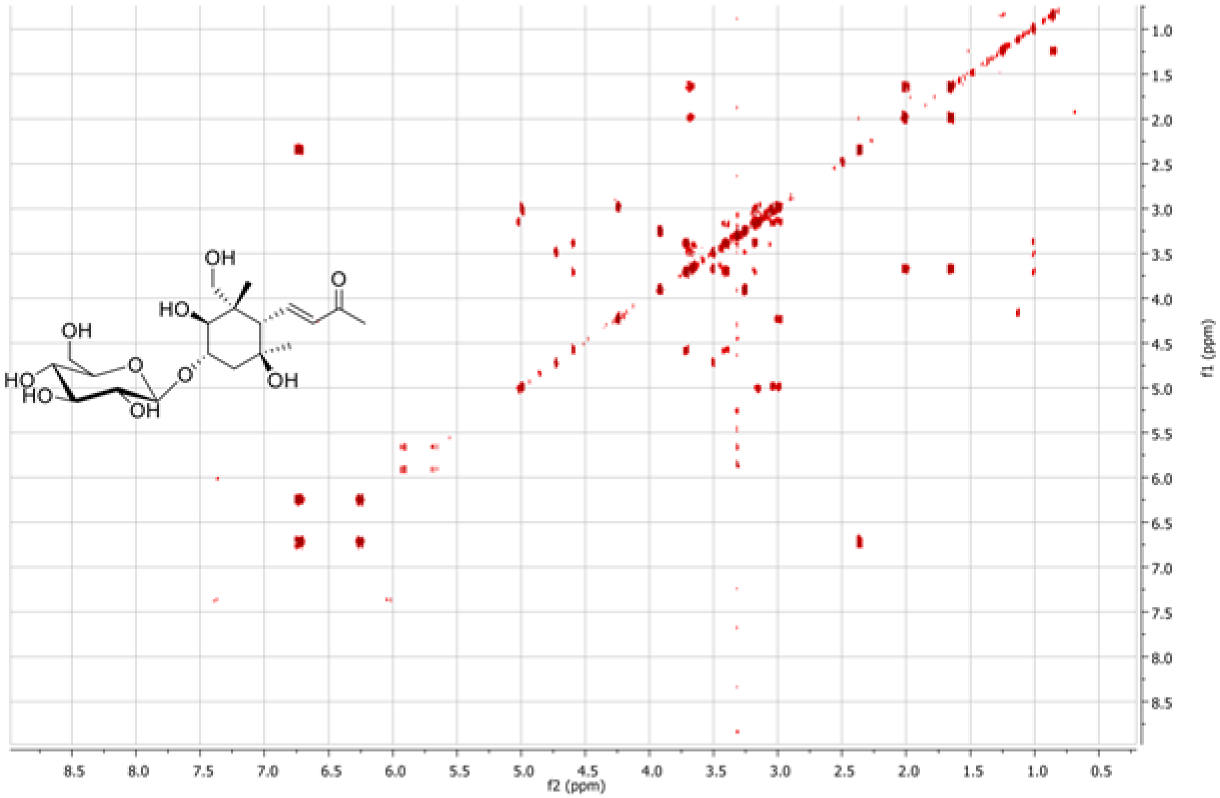

Figure S4. COSY NMR spectrum (DMSO-d_6) of euphocactoside (**5**).

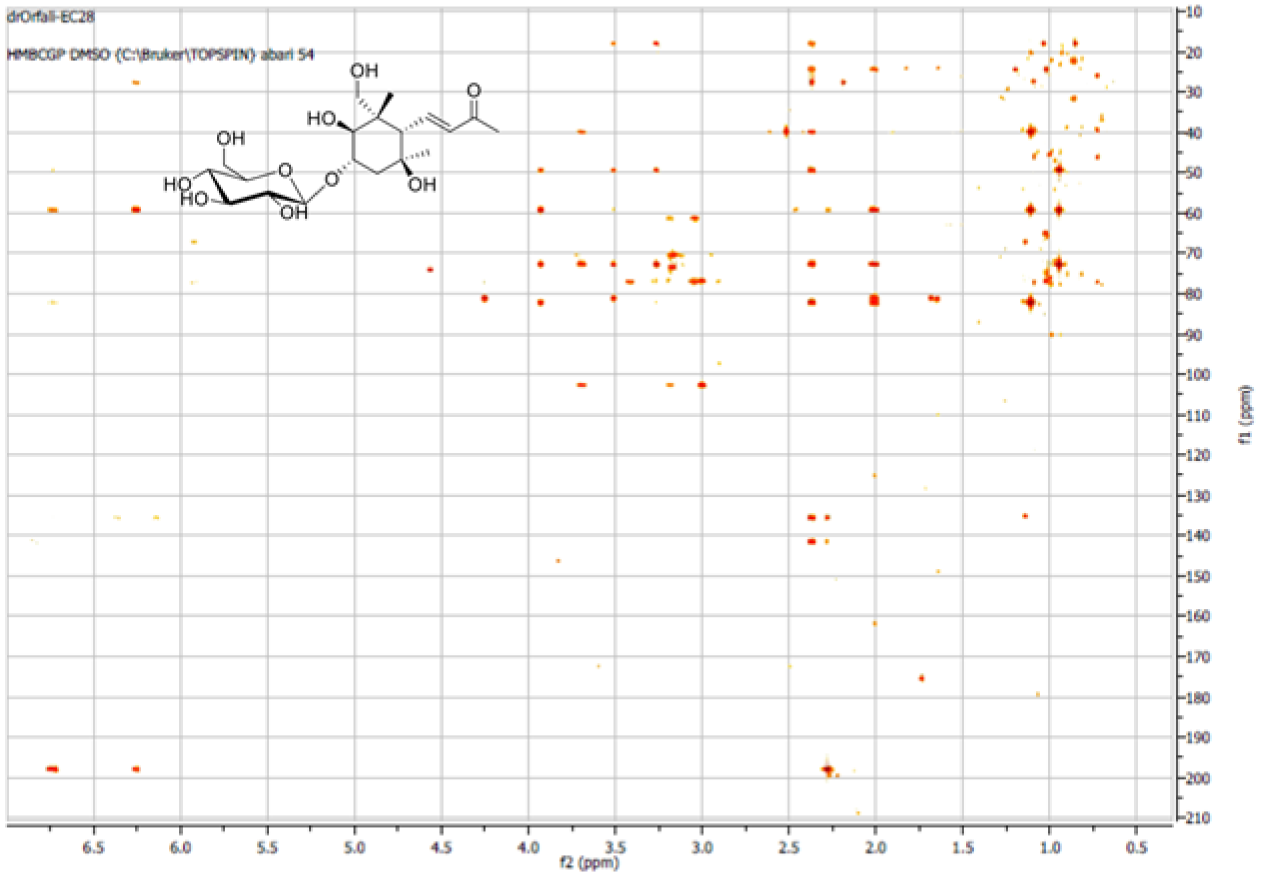

Figure S5. HMBC NMR spectrum (DMSO-d_6) of euphocactoside (**5**).

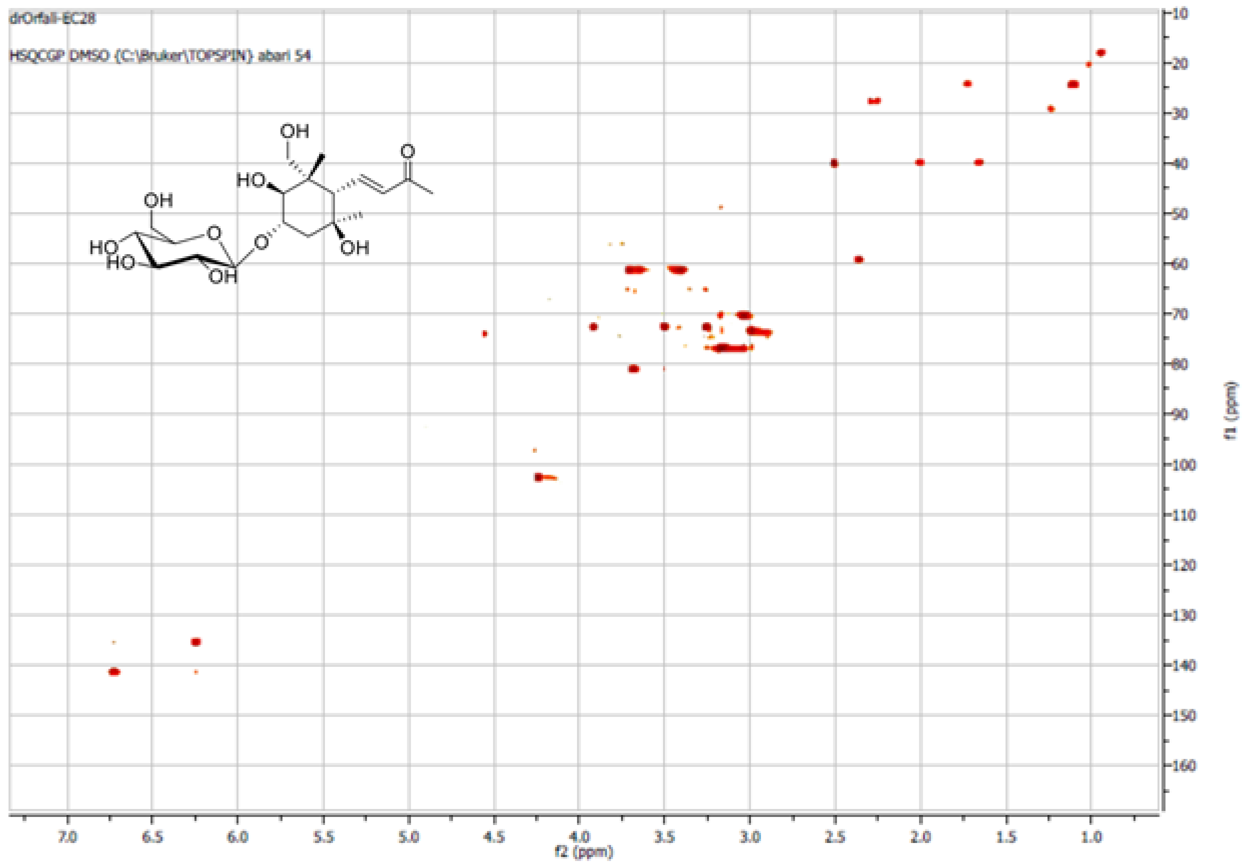

Figure S6. HSQC NMR spectrum (DMSO-d_6) of euphocactoside (**5**).

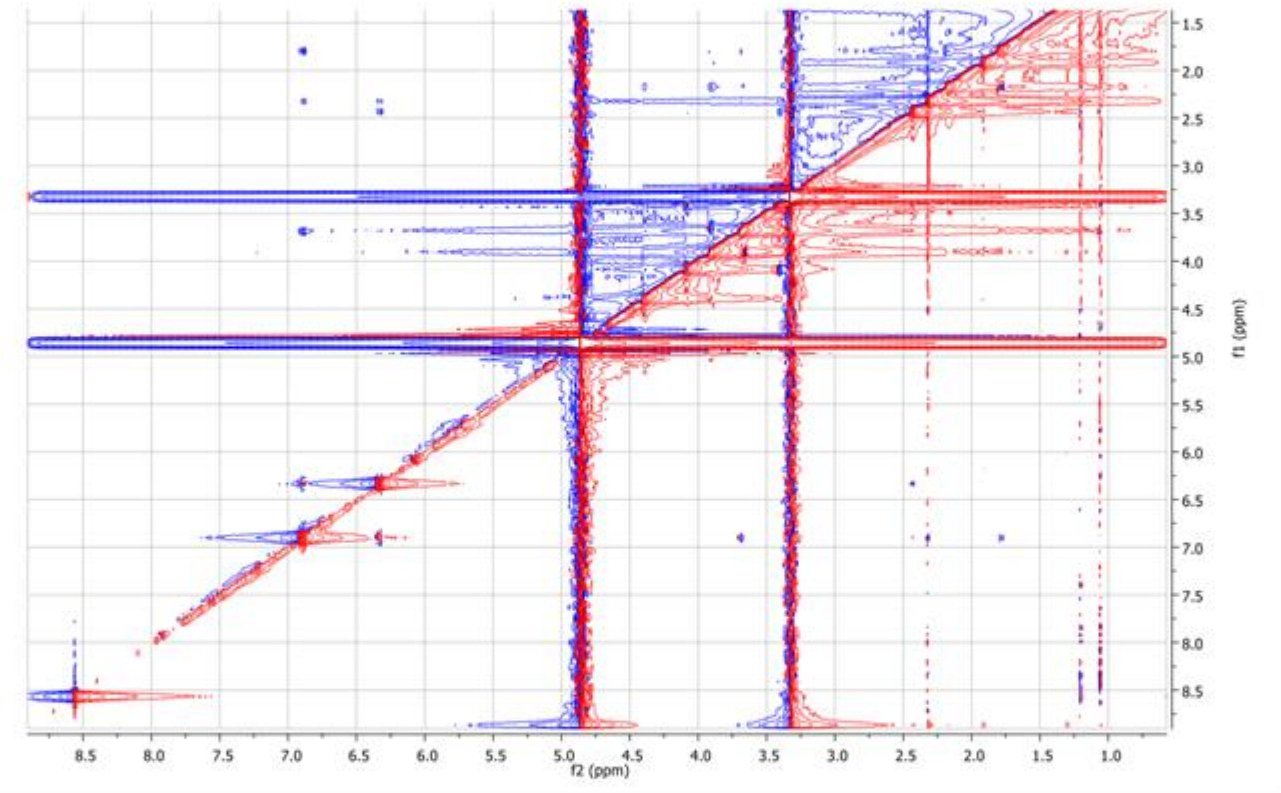

Figure S7. ROESY NMR spectrum (CD_3OD) of euphocactoside (**5**).
